# Comparative analysis indicates that alternative splicing in plants has a limited role in functional expansion of the proteome

**DOI:** 10.1186/1471-2164-10-154

**Published:** 2009-04-09

**Authors:** Edouard I Severing, Aalt DJ van Dijk, Willem J Stiekema, Roeland CHJ van Ham

**Affiliations:** 1 Applied Bioinformatics, Plant Research International, PO Box 16, 6700 AA Wageningen, The Netherlands; 2 Laboratory of Bioinformatics, Wageningen University, Dreijenlaan 3, 6703 HA Wageningen, The Netherlands; 3 Centre for BioSystems Genomics, PO Box 98, 6700 AB Wageningen, The Netherlands

## Abstract

**Background:**

Alternative splicing (AS) is a widespread phenomenon in higher eukaryotes but the extent to which it leads to functional protein isoforms and to proteome expansion at large is still a matter of debate. In contrast to animal species, for which AS has been studied extensively at the protein and functional level, protein-centered studies of AS in plant species are scarce. Here we investigate the functional impact of AS in dicot and monocot plant species using a comparative approach.

**Results:**

Detailed comparison of AS events in alternative spliced orthologs from the dicot *Arabidopsis thaliana *and the monocot *Oryza sativa *(rice) revealed that the vast majority of AS events in both species do not result from functional conservation. Transcript isoforms that are putative targets for the nonsense-mediated decay (NMD) pathway are as likely to contain conserved AS events as isoforms that are translated into proteins. Similar results were obtained when the same comparison was performed between the two more closely related monocot species rice and *Zea mays *(maize).

Genome-wide computational analysis of functional protein domains encoded in alternatively and constitutively spliced genes revealed that only the RNA recognition motif (RRM) is overrepresented in alternatively spliced genes in all species analyzed. In contrast, three domain types were overrepresented in constitutively spliced genes. AS events were found to be less frequent within than outside predicted protein domains and no domain type was found to be enriched with AS introns. Analysis of AS events that result in the removal of complete protein domains revealed that only a small number of domain types is spliced-out in all species analyzed. Finally, in a substantial fraction of cases where a domain is completely removed, this domain appeared to be a unit of a tandem repeat.

**Conclusion:**

The results from the ortholog comparisons suggest that the ability of a gene to produce more than one functional protein through AS does not persist during evolution. Cross-species comparison of the results of the protein-domain oriented analyses indicates little correspondence between the analyzed species. Based on the premise that functional genetic features are most likely to be conserved during evolution, we conclude that AS has only a limited role in functional expansion of the proteome in plants.

## Background

Eukarotyes can produce different mRNAs from a single gene transcript through the process of alternative splicing (AS). Large-scale EST sequencing efforts have revealed that AS is widespread among higher eukaryotes and that it greatly affects their transcriptome complexity, with, for instance, more than 60% of human genes and around 20–30% of plant genes undergoing AS [1-3]. The observation that most AS events occur within the coding region of genes suggests that AS has an important role in the proliferation of an organism's proteome diversity [4,5]. Next to proteome expansion, AS can also serve as a post-transcriptional mechanism for regulating gene expression by producing transcript isoforms that contain a premature termination codon (PTC) which can trigger the decay of the transcript through the nonsense-mediated mRNA decay (NMD) pathway [6].

Although most AS events have the potential to produce different protein isoforms, the extent to which these isoforms are functional is currently unknown. This issue has been addressed using different *in silico *approaches. A number of studies, in which AS isoforms were structurally modeled, have yielded insight into the impact of AS on the stability and function of proteins (see e.g. [7-9]). Structural information, however, is available for only a small fraction of known proteins. An alternative approach has therefore been the comparative analysis of AS between two or more species. The basic assumption behind this type of analysis is that functional genetic features are more likely to be conserved across species than non-functional ones. Those features can be gene-specific, such as the intron/exon structure of a particular gene, or mechanistic and associated with particular cellular processes. In the context of conservation of AS features, the former involve searching orthologous genes for similar AS events that were likely present in the common ancestor of the species under study and that were retained due to an enduring selective advantage of the event. Mechanistic features of a process can often be identified within a single species but if they are also found in a second organism, then their functionality has likely been conserved during evolution.

At the gene-structural level, conserved AS events are identified as similar or distinct event types on orthologous introns and/or exons of at least two species (see for instance [10-12]). However, conserved AS events do not necessarily have similar effects on the encoded proteins while non-conserved AS events on the other hand, may have similar effects on the encoded protein. If the functionalities of more than one isoform are simultaneously favored, then it is the polymorphic character of the gene that provides the selective advantage. Conservation of such polymorphic characters can be identified by searching for homologous alternatively spliced genes in two species for which protein isoforms are modulated in similar ways through AS (see for instance [13]).

A number of general mechanistic features of AS have been identified in diverse species over the past few years. For instance, not only are the fractions of all genes that undergo AS similar in *Arabidopsis thaliana *and rice but also the frequencies of the individual AS events are similar in both species [1,3]. Another conserved mechanistic feature in dicot plant species is the inverse correlation between the UA content of introns and the incidence of AS in these introns [14]. The presence of UA-rich sequences seems to enhance splicing in rice, indicating that such sequences in introns are important for the splicing process [14]. A recent comparative study in which alternatively spliced genes of a wide range of eukaryotes were compared has revealed that genes encoding proteins with particular activities, for instance DNA-, RNA- and calcium-binding, had elevated levels of AS [15]. In line with this study, various protein domains have been shown to be disproportionately distributed towards alternatively spliced genes in humans [16].

Other biases involving the mechanisms by which AS modulates the function of proteins have also been identified in animal species. For instance, AS within the protein coding region of animal genes has been shown to occur more frequently outside than inside the boundaries of predicted functional domains [17]. The authors also reported that evolutionary selection tends to favor AS events that remove entire functional domains. In fact, it has been shown that tissue-specific AS events in mouse transcription factor-encoding genes frequently involve the removal/addition of entire DNA-binding domains [18]. Finally, genome-wide functional profiling of spliced-out domains has revealed that a number of domain types, e.g. protein-binding domains, are more frequently spliced-out by AS than expected by chance [19].

Although it has been suggested that AS in plants has a less prominent role in the expansion of proteome diversity than in animals [20], protein-centered studies that focus on the functional impact of AS in plants are very scarce. In this study, we evaluate the hypothesis that AS in plants contributes to expansion of the functional proteome by analyzing the species *Arabidopsis thaliana*, *Oryza sativa *(rice), *Zea mays *(maize) and *Populus thrichocarpa*. To investigate whether the production of different functional isoforms from a single gene through AS persists during evolution, we analyzed the extent to which polymorphism induced by AS is conserved in orthologous genes. The comparative analysis was performed between the monocot rice and dicot *Arabidopsis *and between the two monocots rice and maize. Following this ortholog-based comparison, we analyzed conservation of more general mechanistic aspects of AS. These analyses were performed separately on each species and the results were compared afterwards. Finally, we address the role of AS in proteome diversification and discuss the evolutionary events that might have led to the patterns that we observed in the plant species.

## Results

### Gene structure and AS predictions

An overview of the gene structure and AS prediction results prior to the construction of hypothetical isoforms is given in Table 1. A surprisingly large number of putative loci were predicted in the maize genome by the methods used here. Unlike the genomic data for *Arabidopsis*, rice and *Populus*, for which a significant part of the genome is represented by few large contigs corresponding to the chromosomes, the genomic data for maize was represented by over 16,000 BAC contigs. For this reason, additional steps were taken for processing the raw maize predictions (see Material and Methods). From all the initially predicted maize loci, only 9360 met our criteria and were considered further.

**Table 1:** Gene structure and AS predictions

	*Arabidopsis*		rice		poplar		maize	
Gene predictions								
Gene structures	36,373		60,431		17,573		173,277	
Loci	25,383		39,252		15,676		140,903	
								
Anchored								
Gene structures	19,949		18,398		4,686		11,147	
Loci	17,754		17,000		4,501		9,360	
Loci with AS	4,338	24.4%	4,703	27.7%	542	12.0%	3,724	39.8%
								
AS event type *								
AA	1,736	23.0%	1,577	16.5%	176	23.3%	1,589	19.1%
AD	932	12.4%	1,017	10.6%	92	12.2%	1,089	13.1%
AP	209	2.8%	590	6.2%	32	4.2%	391	4.7%
IR-CI	3,918	51.9%	4,972	51.9%	302	40.1%	4,072	48.9%
ES-CE	751	10.0%	1,428	14.9%	152	20.2%	1,178	14.2%
Total events	7,546		9,584		754		8,319	

*AA: Alternative acceptor, AD: Alternative donor, AP: Alternative position, IR: Intron retention, CI: Cryptic introns, ES: Exon skipping, CE: Cryptic exons. The frequencies of the individual AS events are derived from a non-redundant set of AS relationships in which the reciprocal event types CI and IR, and ES and CE are grouped into the IR-CI and ES-CE classes, respectively.

AS was detected in 24% and 28% of the tested loci from *Arabidopsis *and rice, respectively. The frequencies of the individual AS event types in these two species are comparable to those that have been reported in a previous genome-wide study [3]. Compared to *Arabidopsis *and rice, the fraction of maize genes that are predicted to undergo AS is relatively high (~40%). However, the distribution of the individual AS events within this species is similar to those found in *Arabidopsis *and rice (Table 1). The lowest number of genes and also the lowest frequency of AS (~12%) were found in poplar (Table 1). These results are likely to be the consequence of the relatively low amount of publicly available transcript data. For this reason, *Populus *was excluded from further analyses.

After constructing hypothetical isoforms for the remaining three species, final sets of gene structures were obtained and comprised 36,950, 40,543 and 25,064 isoform models for *Arabidopsis*, rice and maize, respectively. BlastX searches with the virtual transcripts of the isoforms within this set retrieved an additional 467 *Arabidopsis*, 1,219 rice and 38 maize annotated proteins.

### Alternative splicing in orthologous genes

Out of 10,859 orthologous groups predicted by Inparanoid between *Arabidopsis *and rice, 6,131 main-orthologs had their CDS fully supported by EST evidence. AS was detected in both species for 713 of these ortholog pairs (39.2% of alternatively spliced *Arabidopsis *loci within the ortholog set). Of the 15,951 initial orthologous groups that were predicted between maize and rice, 3,053 main ortholog pairs were kept. From these, 481 pairs (~53% of alternatively spliced rice-genes within this ortholog set) were predicted to undergo AS in both species.

**Figure 1:**
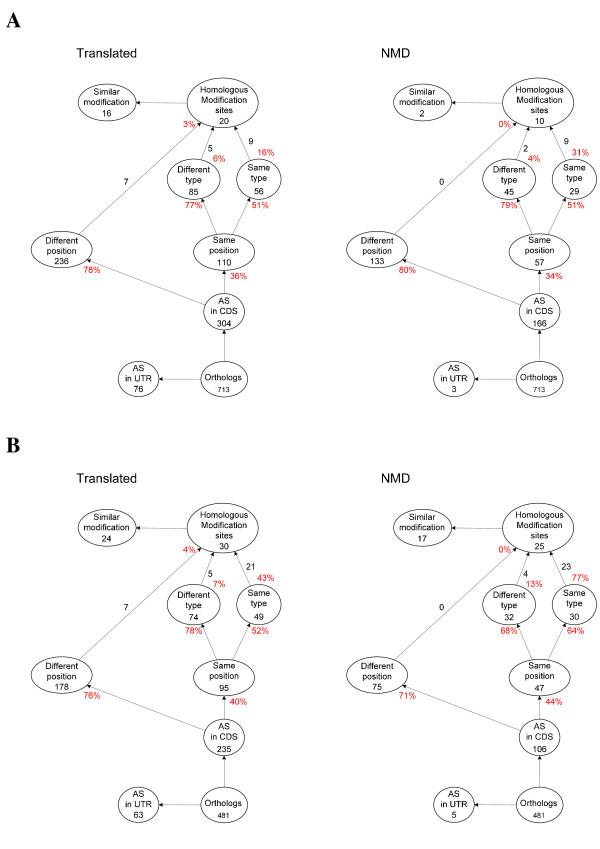
**Dissection of AS events in orthologous genes**. A schematic view is provided of the dissection of AS events in *Arabidopsis*-rice orthologs (A) and maize-rice orthologos (B). The dissection is given for both the translated- and NMD set. Number of orthologous genes within each category is indicated below the name of the category. Numbers next to arrows represent the number of pairs within the category from which the arrow originates that contribute to the number of pairs within the category pointed to by the arrow. For a number of classes the fraction of ortholog pairs from its preceding class that is contained within the child class is provided (in red). Note: several ortholog pairs can be represented in more than one child class as a number of genes have more than one AS variant.

Two sets of alternatively spliced ortholog pairs were constructed: (*i*) ortholog pairs with AS variants that were predicted to be likely targets for the NMD-pathway in both species (NMD-set); and (*ii*) ortholog pairs with AS variants that were predicted to be translated into proteins in both species (translated-set). These two sets were stepwise dissected into sets containing ortholog pairs with (Figure 1): (*i*) AS events on different positions; (*ii*) AS events on the same position; (*iii*) different type of events on the same position; (*iv*) the same type of event on the same position; (*v*) homologous modification sites; and (*vi*) similar modifications (see Additional file 1 and Additional file 2).

The number of cases with the same type of AS event in orthologous introns between *Arabidopsis *and rice was low in both the translated and the NMD sets (Figure 1a), in agreement with results from previous studies [3,14]. A total of 16 ortholog pairs (~2.2%) from the translated set, including a pair of previously studied MYB transcription factors [12], were found to have similar modifications to the principle protein isoform as the result of AS. In contrast, only two such cases were found in the NMD set. The fraction of ortholog pairs with AS in the CDS region that had AS events on different positions were roughly the same in both the NMD- and translated sets. The same was true for the fraction of ortholog pairs with the same and different type of events on orthologous introns.

In total 24 (~4.9%) and 17 (~3.5%) maize-rice ortholog pairs had similar modifications in the translated and NMD set, respectively (Figure 1B). Although these numbers are higher than those observed for the *Arabidopsis*-rice orthologs, they are still low. Within the translated set of the maize-rice orthologs, the distributions of AS events on different positions and same- and different type of events on orthologous intron positions were not too different from the same distributions observed in both the NMD- and translated set of the *Arabidopsis*-rice orthologs. Within the NMD-set of the maize-rice orthologs, the number of different AS event-types on orthologous introns was roughly the same as the number of similar AS events on orthologous introns. In all subsets, similar AS events on orthologous positions were the greatest contributors to the subset of similar modifications. The GO terms corresponding to the ortholog pairs within the different subsets were analyzed but no clear overrepresentation of any particular term could be identified (data not shown).

### Disproportionately distributed protein domains

We investigated whether particular protein domains were disproportionately distributed towards either constitutively (CS) or alternatively (AS) spliced genes in all three plants. Disproportionately distributed domains are indicative for the under- or overrepresentation of AS in genes with a particular function. To this end, the distributions of 2,626 domain types in *Arabidopsis*, 2,487 in rice and 1,791 in maize were analyzed. A domain was considered to be disproportionately distributed when the ratio AS- to CS-genes encoding the domain was significantly different from the same ratio for all genes that lack that particular domain. The fraction of domains that are disproportionately distributed in the studied species (Table 2) ranges from 0.2% to 0.3%. Four domain types were found to be disproportionately distributed in all three species. Three of these, the pentatricopeptide (PF01535), the UPD-gluccuronosyl/UDP-glucotransferase (PF00201) and the Cytochrome P450 (PF00067) domains were overrepresented in CS genes. The fourth domain, the RRM_1 domain (PF00076), which is an RNA recognition motif, was the only domain that was significantly overrepresented in AS genes of all the species studied.

**Table 2:** Disproportionately distributed domains

Species	Pfam Id	Domain name	CS genes w. domain	AS genes w. domain	p-value	Bias
*Arabidopsis*						
	PF00560	LRR_1	153	14	3.55E-08	CS
	PF08263	LRRNT_2	123	9	5.65E-08	CS
	PF02519	Auxin_inducible	55	0	1.44E-07	CS
	PF01535	PPR	146	15	4.03E-07	CS*
	PF00076	RRM_1	114	77	1.61E-06	AS*
	PF00201	UDPGT	69	3	2.73E-06	CS*
	PF00067	p450	143	18	1.03E-05	CS*
	PF00847	AP2	115	15	0.0001159	CS
rice						
	PF01535	PPR	179	17	1.53E-11	CS*
	PF00076	RRM_1	99	89	1.04E-08	AS*
	PF00646	F-box	202	34	4.69E-07	CS
	PF00067	p450	137	19	1.52E-06	CS*
	PF00201	UDPGT	70	5	3.59E-06	CS*
	PF07859	Abhydrolase_3	28	0	0.000101	CS
maize						
	PF01609	Transposase_11	52	0	9.62E-13	CS
	PF01535	PPR	64	3	5.70E-12	CS*
	PF00201	UDPGT	46	7	9.53E-06	CS*
	PF00067	p450	46	10	0.0001739	CS*
	PF00076	RRM_1	47	65	0.0002	AS*

* Domains that have the same distribution bias in all three species. The distribution values and bias are shown for all domains that were significantly overrepresented in either constitutively (CS) or alternatively (AS) spliced genes.

### Intron associated with protein domains

To determine whether introns located within predicted Pfam domains are less or more frequently subjected to AS compared to other introns within the CDS regions of genes, the ratio between AS intron clusters and CS intron clusters within the predicted domains was compared to the same ratio for intron clusters located outside predicted domains. In *Arabidopsis *2,486 (~6%) of the 41,107 intron clusters located within the boundaries of predicted Pfam domains and 1,941 (~7.6%) of the 25,657 clusters outside Pfam domains were found to be AS clusters. Similarly, in rice, 2,487 (~7%) of the 35,595 intron clusters within domains and 2,088 (~9%) of the 23,093 clusters outside predicted domains were AS clusters. The same trend was also observed in maize where 2,292 (~14%) of the 16,466 intron-clusters within predicted domains were AS-clusters, while 1,634 (~19%) of the 8,677 intron clusters outside predicted domains were AS clusters. The difference between the AS-cluster frequencies within and outside predicted domains is significant in all three species (p-value << 0.01).

Next, we investigated whether particular protein-domain types have retained a higher propensity for AS during evolution by having particular sequence or structural features. To this end, the ratio between AS and CS intron clusters located within a particular domain type was compared to the same ratio between intron clusters located within all other domains types. A total of 304, 309 and 316 domain types in *Arabidopsis*, rice and maize, respectively, were excluded from the test as no introns were found within their boundaries. The number of domain types that were enriched with either AS or CS introns ranged from six in maize to twelve in *Arabidopsis *(Table 3). The Kinesin domain (PF00225) in *Arabidopsis *was the only domain that was significantly enriched with constitutively spliced introns; no domain types enriched with CS intron clusters were detected in rice and maize. Not a single domain type was found that was enriched for AS intron clusters in all species.

**Table 3:** Intron/domain associations

Species	Pfam Id	Domain name	CS Introns	AS introns	p-value	Bias
*Arabidopsis*						
	PF00303	Thymidylat_synt	0	14	2.20E-16	AS
	PF00724	Oxidored_FMN	8	9	1.76E-07	AS
	PF06487	SAP18	0	5	8.32E-07	AS
	PF04628	Sedlin_N	2	6	1.27E-06	AS
	PF00697	PRAI	1	5	4.74E-06	AS
	PF00225	Kinesin	228	1	8.68E-06	CS
	PF07847	DUF1637	12	8	1.21E-05	AS
	PF03226	Yippee	13	8	1.85E-05	AS
	PF00582	Usp	55	14	5.99E-05	AS
	PF01716	MSP	1	4	6.52E-05	AS
	PF01789	PsbP	12	7	8.07E-05	AS
	PF07172	GRP	4	5	8.53E-05	AS
rice						
	PF00234	Tryp_alpha_amyl	0	11	2.33E-13	AS
	PF00504	Chloroa_b-bind	14	12	6.19E-08	AS
	PF00230	MIP	32	15	4.94E-07	AS
	PF02913	FAD-oxidase_C	1	6	8.50E-07	AS
	PF04172	LrgB	0	5	1.82E-06	AS
	PF05564	Auxin_repressed	2	5	3.38E-05	AS
	PF00011	HSP20	3	5	8.49E-05	AS
maize						
	PF01559	Zein	0	7	1.07E-06	AS
	PF01040	UbiA	18	14	4.36E-05	AS
	PF01596	Methyltransf_3	0	5	5.45E-05	AS
	PF03763	Remorin_C	7	9	9.41E-05	AS
	PF00297	Ribosomal_L3	5	8	9.96E-05	AS
	PF00722	Glyco_hydro_16	10	10	0.0001406	AS

An overview is given for all domains in *Arabidopsis*, rice and maize with a significant excess of either constitutive (CS) or alternative (AS) intron clusters.

### Spliced-out protein domains

The function of a protein can be modulated by AS through the addition or deletion of complete functional domains but also of small stretches of amino acids. However, because it is impossible to assess the impact of small amino acid sequence changes on protein function without additional structural or experimental data, only AS events were considered that removed whole protein domains while leaving at least one domain intact. A total of 936, 976 and 1,211 alternatively spliced loci with at least one isoform encoding a multi-domain protein were analyzed in *Arabidopsis*, rice and maize, respectively.

In total, 218 (117 types), 244 (131 types) and 148 (88 types) spliced-out domains were identified in *Arabidopsis *(see Additional file 3), rice (see Additional file 4) and maize (see Additional file 5), respectively. Twenty-nine domain types were found to be spliced out in all three species, 28 of which (Table 4) were located on at least two different genomic regions in at least two of the three species. We tested whether particular domains are spliced-out at significantly elevated rates within the subset of isoforms that were analyzed for spliced out domains. Only the PF00098 (zf-CCHC) domain was spliced out at significantly elevated rates in both maize and *Arabidopsis *compared to all other spliced out domains (Fisher exact test p-values: 8.4e-05 in *Arabidopsis *and 0.001 in maize). No domain type was found that was spliced out with significantly elevated rates compared to other domains in all three species.

**Table 4:** Number of shared spliced-out domains

Pfam Id	Domain name	*A. thaliana*	rice	maize
PF00400	WD40	15	10	7
PF00023	Ank	9	5	-
PF00076	RRM_1	10	11	4
PF02798	GST_N	3	3	3
PF00153	Mito_carr	3	6	3
PF01357	Pollen_allerg_1	-	3	3
PF03765	CRAL_TRIO_N	2	3	2
PF00270	DEAD	2	3	-
PF00226	DnaJ	3	-	2
PF00043	GST_C	3	2	3
PF07646	Kelch_2	2	-	3
PF00560	LRR_1	6	9	2
PF00072	Response_reg	2	2	-
PF00627	UBA	-	2	2
PF08240	ADH_N	1	3	4
PF00892	DUF6	6	1	3
PF08263	LRRNT_2	3	7	1
PF00249	Myb_DNA-binding	10	1	5
PF00614	PLDc	2	1	2
PF01535	PPR	7	3	1
PF00085	Thioredoxin	1	2	2
PF00035	Dsrm	2	1	2
PF00036	Efhand	11	10	1
PF00643	zf-B_box	2	2	1
PF00096	zf-C2H2	1	2	4
PF00642	zf-CCCH	1	6	4
PF00098	zf-CCHC	6	1	6
PF00641	zf-RanBP	2	1	2

The top five assignments of molecular function-related GO terms [21], accounting for around half of all assignments to the spliced-out domains in *Arabidopsis *and rice, were: nucleic acid binding (GO:0003676), zinc ion-binding (GO:0008270), calcium ion-binding (GO:0005509), protein-binding (GO:0005515) and ATP binding (GO:0005524) (for *Arabidopsis *and rice, respectively see Additional file 6 and Additional file 7). In maize, around 45% of GO term assignments were nucleic acid binding and zinc ion-binding (see Additional file 8)

Next, the splicing patterns of the spliced-out domains and their resulting domain architectures (products) were analyzed and compared between the species. A total of 152 unique splicing patterns resulting in 129 unique products were identified in *Arabidopsis*. In rice these numbers were 174 and 151, respectively. In maize, a total of 103 patterns leading to 92 products were identified. Cross-species comparison resulted in the identification of 14 splicing patterns and 20 products that were shared by all three species.

Closer inspection of the splicing patterns revealed that 65 (~42.8%) of the *Arabidopsis*, 66 (~37.9%) of the rice and 43 (41.7%) of the maize splicing patterns involved the removal of a domain which was a unit of a tandem-repeat (for *Arabidopsis*, rice and maize, respectively see Additional file 9 and Additional file 10 and Additional file 11). Fifteen domain types were identified that were at least once removed from a tandem repeat in all three species (Table 5). Eight out of the 14 (57%) splicing patterns that were shared between the three species involved the removal of a domain from a tandem repeat.

**Table 5:** Number of splicing patterns of domain types spliced-out from a tandem repeat in all three species

Pfam Id	Domain name	*A. thaliana*	rice	maize
PF00076	RRM_1	4	5	4
PF00400	WD40	9	6	4
PF00153	Mito_carr	2	3	2
PF00035	dsrm	2	1	1
PF00036	efhand	5	5	1
PF00096	zf-C2H2	1	1	1
PF00249	Myb_DNA-binding	2	1	2
PF00412	LIM	1	1	2
PF00614	PLDc	1	1	1
PF00641	zf-RanBP	1	1	2
PF00642	zf-CCCH	1	2	2
PF00643	zf-B_box	1	1	1
PF00892	DUF6	2	1	1
PF01535	PPR	5	1	1
PF06943	zf-LSD1	1	1	1

## Discussion

In this study we addressed the functional impact of alternative splicing in plants using a comparative analysis approach. According to the ascribed role of AS as a mechanism for expanding proteome diversity, a gene can become polymorph in its expression through an additional splice variant that encodes a different, yet functional protein. When the principle and alternative protein isoforms each confer a selective advantage, both are likely to be retained during evolution. This can be achieved through either retention of the AS-induced polymorphism or through gene-duplication followed by sub-functionalization of the duplicates [22]. Not only different protein isoforms but also AS-mediated regulation of gene expression through the production of PTC containing isoforms that are destined to be degraded by the NMD-pathway can provide a selective advantage. The exact AS event is not necessarily required to remain the same for both the production of protein isoforms and the production of PTC containing transcripts isoforms. In this study, we investigated to what extent AS induced polymorphism as well as AS-mediated gene-regulation are conserved between orthologous genes of the dicot *Arabidopsis *and the monocot rice and orthologous genes of the two monocots maize and rice. The comparison was focused on the functional outcome of the AS events rather than on the AS events themselves. The number of cases in which orthologs in *Arabidopsis *and rice contained AS variants that are likely targets for NMD was roughly half that of ortholog pairs with variants that can be translated into proteins. Only a very small number of cases were found in which AS events in both orthologs resulted in similar modifications to the principle protein product. Interestingly, the fractions of ortholog pairs that have AS events on different positions and those that have similar or different event types on the same position were quite similar in both the NMD and translated subsets of the *Arabidopsis*-rice orthologs. Because the isoforms in the NMD subset would never function as protein, this similarity suggests that a considerable fraction of the putatively conserved AS events are not the result of functional conservation. Similar values for the distributions mentioned above were observed in the translated subset of the maize-rice orthologs. This similarity further strengthens the notion that conservation of AS events is not the result of the preservation of functional AS induced protein polymorphism. Although the fraction of orthologs that have similar AS-event types on orthologous intron-positions leading to modifications at similar sites is higher in the maize-rice comparison than that observed for the *Arabidopsis*-rice orthologs in the translated as well as the NMD set, these numbers are still remarkably low. In the NMD subset of the maize-rice orthologs the fractions of different- and similar event types on orthologous intron-positions are very similar. In summary, the results suggest that conservation of AS events as the result of the selective advantage of retaining the ability to produce multiple protein isoforms through AS is not frequent, even at short evolutionary distances.

Previous studies on animal data (see introduction) have revealed similarities between genes undergoing AS in various species. The observed patterns are not the result of ortholog-level conservation, which is evident from the low numbers of conserved AS events between, for instance, human and mouse [23]. However, the patterns do point to the existence of mechanistic features that are associated to- or reflected in the AS process. One such mechanistic feature in animals is the high frequency of exon skipping events which is the consequence of the exon definition mechanism through which splice-sites are recognized [24]. In contrast, the high frequency of intron retention events in plants is the consequence of the intron definition mechanism through which many plant introns are recognized [25].

In contrast to animals, protein function-oriented studies of genome-wide AS patterns in plants are scarce. Motivated by the results obtained from computational analysis of the impact of AS on protein function in animals, we have applied a number of those analyses on plant data. The first analysis involved the distribution of Pfam domains over constitutively and alternatively spliced genes using a similar method as previously performed on human data [16]. Three domains were significantly underrepresented in alternatively spliced genes in all three species. Genes encoding these domains share at least two properties that have previously been shown to be correlated with AS frequencies. First, the genes belong to highly expanded gene families in plants [26-28]. Recent studies in animal species have shown that the incidence of AS is inversely correlated to gene family size [22,29,30].

However, such an inverse correlation has not been found in *Arabidopsis *and rice [31]. These conflicting results may be the consequence of the different methods that have been used for delineating gene families. Second, genes encoding these domains have relatively low average numbers of introns in all three species (data not shown). It has previously been shown that the incidence of AS is positively correlated with the number of introns [15].

Only one domain, the RRM domain, was significantly overrepresented in alternatively spliced genes in all species. This RNA recognition domain, like the domains mentioned above, is found in a large number of genes, many of which are involved in the regulation of the splicing process [32]. However, in contrast to the three domains that were underrepresented, the average number of introns harbored by genes encoding the RRM domain is higher than the average number of introns in all genes in all three species and this might in part explain the observed data (data not shown).

Zooming in on the sequence level, it has been shown in four animal species that AS occurs less frequently within than outside the boundaries of predicted protein domains [17]. We observed a similar trend in all three plant species. This result, together with those presented by Kriventseva and coworkers [17], suggests that in general AS events within the boundaries of protein domains are less favored in evolution.

It has been reported that domains with particular functions are more frequently targeted by AS than expected by chance (reviewed in [33]). Not only the location of introns but also the location of AS events within a gene are to a certain degree related to sequence- and structural features of the encoded protein [34,35] and it was hypothesized that particular domains are more prone to undergo AS within their boundaries as the result of such features. This possibility was tested by searching for domains that were significantly enriched with alternatively spliced rather than with constitutively spliced introns. Although a few domains were identified that were significantly enriched with alternatively spliced introns in individual species, none of such domains were found across all species. This result suggests that none of the studied domains has a detectable evolutionary propensity for undergoing AS within its boundaries.

The final analysis involved AS events that remove entire protein domains. The specific AS events that lead to the removal of the domains are not required to be located within the boundaries of the spliced-out domain. In human genes, various domains have been shown to be more often spliced-out than average [19]. Here, we identified a number of cases in which AS events resulted in the removal of complete protein domains in all three plant species, but only one domain was found to be spliced out at a significantly elevated rate in both *Arabidopsis *and maize. The top five molecular function-related GO terms of the spliced-out domains, comprising around 50% of all GO-term assignments, were the same in *Arabidopsis *and rice while in maize only two of these five GO-terms comprised 45% of GO term assignments. Genes having these GO terms in *Arabidopsis *have been shown to have elevated levels of AS [36]. However, in that study only the AS levels of genes with nucleotide binding activities were found to be significantly elevated.

Interestingly, in a large fraction of the unique splicing patterns in all species, the spliced-out domain was a unit of a tandem repeat. A similar observation has been reported in a study that was conducted on data derived from the Swiss-Prot and PDB- databases [7]. The authors suggested that AS within repeated regions of a protein is more likely to be tolerated. Their conclusion is supported by the observation that variation in repetitive regions has frequently been used in evolution for modifying the properties of proteins without invoking loss of their fold [37]. It is also possible that the large fraction of domains that are spliced-out from tandem repeats is the consequence of the duplication events themselves as is seen for the frequent appearance of AS through tandem exon duplications [38]. However, further research is needed to clarify the observed frequent removal of domains from a tandem repeat.

## Conclusion

Although a considerable number of functional AS events in plants have been described in literature [5], we have shown here that only a small number of AS events have conserved effects at the protein sequence level in *Arabidopsis *and rice. The same has been shown for ortholog genes of the more closely related species maize and rice. The persistence of gene-specific function expansion has thus been very limited over the divergence time between not only the monocot rice and the dicot *Arabidopsis *but also between the two monocots rice and maize. Functional diversification through AS within protein domains appeared to be independent on the nature of the domain itself. The identification of domains that are frequently spliced out in both species is likely to be the result of their abundance rather than their specific function. Taken together, these results, in the context of comparative analysis, suggest that the role of AS as a mechanism for expansion of function proteome diversity in plants is very limited. It has previously been suggested that the majority of AS variants are unlikely to be functional [8,39]. However, as long as such non-functional isoforms are selectively neutral, they can still serve as templates for further diversification and eventually lead to the acquisition of new functions. Their propagation might be enhanced by the NMD-pathway which protects them from being selected against [40]. In conclusion, AS seems to be more a mechanism that leads to functional diversification over evolutionary time, comparable with e.g. gene duplication, than a mechanism for providing an organism during its life span with more functional proteins than the number of genes in its genome.

## Methods

### Data sources and gene structure predictions

Over 1,000,000 transcript sequences for *Arabidopsis*, rice and maize and around 90,000 transcript sequences for poplar were downloaded from the PlantGDB database [41] and inspected using the SeqClean package downloaded from [42]. The (pseudo-)chromosome sequences and annotation data versions TIGR 5.0 and TAIR 7.0 were used for rice and *Arabidopsis*, respectively. For maize, BAC contigs and annotation data were download from the maize sequence ftp site [43]. The chromosome contigs and annotation data for *Populus *version 1.1 were downloaded from the JGI poplar download-site [44]. Annotations sets of maize and *Populus *were expanded by adding proteins from these organisms that were extracted from NCBI Nr database [45]. The most likely genomic origin of each transcript was determined by performing blastN searches [46] (word size = 32; e-value threshold = 1e^-10^). Only those transcripts for which ≥90% of their bases could be matched to the genome with a mean identity ≥97% were considered further. Clusters of transcripts that mapped to overlapping genomic regions were realigned to the genome using the program GeneSeqer [47] which has specific splice-site models for each species. Only exons that were predicted with an alignment score ≥0.98 were considered further. As the maize genome, unlike the genomes of the three other species, is represented by more than 16,000 BAC contigs, only one copy was considered from the initially predicted loci that had exactly the same set of gene structures. Gene structures encoding annotated proteins were identified through blastX searches with their virtual transcripts against the corresponding predicted proteome of the species. Only those loci containing at least one gene structure with an exact match to an annotated protein were considered further.

### Prediction of AS and construction of isoforms

The majority of the available transcript data for *Arabidopsis *and rice is represented by ESTs. To overcome the shortage of full-length cDNA sequences, additional hypothetical full-length isoforms were constructed using a previously described method [48] with the following modifications. In our method, only those isoforms that fully encoded an annotated protein were used as reference isoforms. AS events were identified by comparing these reference isoforms to all other isoforms (second isoform) from the same locus for which either of the following conditions were met: (*i*) the two isoforms shared at least one intron or exon; or (*ii*) fifty percent of the exonic nucleotides were shared between both isoforms. The following types of AS events were classified (using terms different from those in [48]): alternative donor (AD), alternative acceptor (AA) and alternative position (AP) events were identified as overlapping introns differing in their donor-, acceptor- or both splice sites, respectively (Additional file 1, A). Intron retention (IR) events were identified as introns in the reference isoform that were fully contained within an exon of the second isoform (Additional file 1, B) and the reciprocal event, cryptic intron (CI), involved an intron in the second isoform that was fully contained within an exon of the reference isoform. Exon skipping (ES) events were identified as internal exons in the reference isoform that were fully contained within an intron in the second isoform (Additional file 1, C) and the reciprocal event, cryptic exons (CE), involved exons in the second isoform that were fully located within an intron of the reference isoform. The outermost 3'- and 5'- genomic boundaries of a hypothetical isoform only differed from those of its reference isoform when the first or last exon of either isoforms was fully located within the first or last intron of the other isoform.

Two open reading frames (ORFs) were assigned to those isoforms that did not fully match to an annotated protein: (*i*) the longest ORF from start to stop that overlaps with the ORF of the reference isoforms and; (*ii*) if possible the ORF starting at the same genomic position as the ORF of the reference isoform. All isoforms containing a termination codon more than 55 nt upstream of the last exon/exon junction were considered as putative targets for the NMD-pathway [49]. The impact of AS on the primary protein sequence encoded by the reference isoform was analyzed by comparing the ORFs of each reference/hypothetical isoform pair at the gene level.

### Comparison of AS in orthologous genes

Orthologs were identified using the program Inparanoid version 1.35 [50]. Needleman-Wunsch alignments [51] were constructed for all possible ortholog pairs using the program Needle from the EMBOSS package version 4.0 [52]. In a number of orthologous groups in which multiple proteins in one of the species were classified as orthologs, the protein pair with the highest global identity was kept. In those cases were multiple orthologs were predicted in both species, all pairs with the bidirectional highest global alignment identity were kept. The final ortholog set only contained protein pairs that were fully supported by EST/cDNA evidence in both species.

As explained above, the classification of some AS events depends on which isoform is chosen as the reference. One commonly used method is to choose the isoform with highest amount of transcript evidence as the reference isoform [53]. The choice can also be based on the evolutionary conservation of either the exon/intron structure of transcript isoforms or the protein isoforms [54]. In the current comparative analysis, the transcript isoforms corresponding to the orthologous proteins were chosen as the reference isoforms.

Comparative analysis of AS patterns at the gene structural level involved searching for conserved introns within the coding regions (CDS) that were predicted to be alternatively spliced in both species. To achieve this, the introns located within the CDS underlying the aligned orthologous proteins were projected onto their global alignment. Conserved introns were subsequently identified as "aligned" introns with the same phase or as "unaligned" introns with the same phase within a distance of six amino acids that could unambiguously be matched.

The second level of comparison involved the identification of similar changes to the orthologous protein sequences resulting from AS events. Three types of similar changes were investigated: (*i*) similar additions were identified by searching for identical insertion sites and comparing the stretches of amino acids; (*ii*) similar substitutions caused by frame shifts were identified by searching for aligned amino acids that were substituted in AS isoforms in both species and comparing the stretches of amino acids resulting from the frame shift(s); (*iii*) similar deletions were detected by searching for stretches of aligned amino acids that were deleted as the results of AS in both species. These changes at identical positions were designated "homologous modification sites". Both (*i*) and (*ii*) were designated "similar modification" if the similarity of the aligned stretches was over 40%.

### Comparison of mechanistic patterns

In contrast to cross species comparison of AS induced polymorphisms, the comparison of mechanistic patterns was not restricted to orthologous gene pairs. The InterProScan program [55] version 4.3.1 was used for the identification of Pfam domains version (21.0) [56] in the deduced protein sequence of each isoform. Only domains that were predicted with an e-value < 0.01 were considered.

The identification of domains that were disproportionately distributed towards either constitutively (CS) or alternatively spliced (AS) genes was performed as described elsewhere [16], with the exception that the *Holm's *correction method [57] was used in the present study for controlling false discovery rates.

Prior to testing whether particular domains were significantly enriched with either constitutively or alternatively spliced introns, clusters of overlapping introns were constructed. All clusters containing more than one unique intron and singleton introns that were retained in one or more isoforms were labeled as alternative splicing (AS) clusters. All other singleton introns were labeled as constitutive (CS) clusters. This clustering step prevents a bias in the results caused by over-counting of individual AS introns (with the exception of CI and RI events, all other events involve at least two AS introns). Fisher exact tests (with Holm's correction method) were performed for each domain type to test whether the ratio between its number of associated AS and CS introns deviated significantly from the ratio between all AS and CS introns located within the boundaries of all domains other than the one being tested.

A spliced-out domain [19] was identified as a domain that was present in only one of the isoforms of a reference/hypothetical isoform pair. The detection of spliced-out domains was only performed on pairs of isoforms that were not predicted to be likely targets for the NMD-pathway.

## Authors' contributions

ES designed the study, performed the analysis and drafted the manuscript. AvD participated in the analysis and preparation of the manuscript. WS supervised the study. RvH initiated and supervised the study and revised the manuscript. All authors read and approved the final manuscript.

## Supplementary Material

Additional file 1 **Detection of AS events and construction of full-length isoforms**. The figure is a schematic representation of the methods used to detect alternative splicing events and to construct full-length isoforms. Click here for file

Additional file 2 **Dissection of AS events in orthologous genes**. The figure is a schematic representation of the method used to dissect AS events in orthologous genes. Click here for file

Additional file 3 **Spliced out domains in *Arabidopsis***. The table provides an overview of the domain types that were found to be spliced out in *Arabidopsis*. Click here for file

Additional file 4 **Spliced out domains in rice**. The table provides an overview of the domain types that were found to be spliced out in rice. Click here for file

Additional file 5 **Spliced out domains in maize**. The table provides an overview of the domain types that were found to be spliced out in maize. Click here for file

Additional file 6 **Molecular function related GO term assignments in *Arabidopsis***. The table provides an overview of the molecular function related GO terms belonging to the spliced out domains in *Arabidopsis*. Click here for file

Additional file 7 **Molecular function related GO term assignments in rice**. The table provides an overview of molecular the function related GO terms belonging to the spliced out domains in rice. Click here for file

Additional file 8 **Molecular function related GO term assignment in maize**. The table provides an overview of the molecular function related GO terms belonging to the spliced out domains in maize. Click here for file

Additional file 9 **Spliced-out domains from tandem repeat units in *Arabidopsis***. The table provides an overview of the spliced out domains in *Arabidopsis *that are a unit of a tandem repeat. Click here for file

Additional file 10 **Spliced-out domains from tandem repeat units in rice**. The table provides an overview of the spliced out domains in rice that are a unit of a tandem repeat. Click here for file

Additional file 11 **Spliced-out domains from tandem repeat units in maize**. The table provides an overview of the spliced out domains in maize that are a unit of a tandem repeat. Click here for file
