# Supplementary material for: Comparative analysis indicates that alternative splicing in plants has a limited role in functional expansion of the proteome
Source: BMC Genomics. 2009 Apr 9;10:154. doi: 10.1186/1471-2164-10-154 (PMC2674458; doi:10.1186/1471-2164-10-154)
Supplement: Additional file 1 — Detection of AS events and construction of full-length isoforms. The figure is a schematic representation of the methods used to detect alternative splicing events and to construct full-length isoforms. [file 1471-2164-10-154-S1.doc]

**AS Detection and isoform construction**.

**Additional file 1. Detection of AS events and construction of full-length isoforms. A**. Alternative donor (AD) events are identified as two overlapping introns (red), one on each isoform, that differ in their donor sites. In the hypothetical isoform, the intron on the reference isoform is substituted by the overlapping intron on the second isoform. **B**. Intron retention (IR) events are characterized as an intron on the reference isoform (red) which is fully contained within an exon on the second isoform. The hypothetical isoform, when compared to the reference isoform lacks the retained intron. **C**. Exon skipping (ES) events are identified as two consecutive introns (red) on the reference isoform that overlap with a single intron on the second isoform (red). In the hypothetical isoform the consecutive introns of the reference isoform are substituted by a single intron of the second isoform.
