# Supplementary material for: Comparative analysis indicates that alternative splicing in plants has a limited role in functional expansion of the proteome
Source: BMC Genomics. 2009 Apr 9;10:154. doi: 10.1186/1471-2164-10-154 (PMC2674458; doi:10.1186/1471-2164-10-154)
Supplement: Additional file 2 — Dissection of AS events in orthologous genes. The figure is a schematic representation of the method used to dissect AS events in orthologous genes. [file 1471-2164-10-154-S2.doc]

Dissection of AS events in orthologous genes

**Additional file 2. Dissection of AS events in orthologous genes**. A) Orthologous exon and introns within the CDS region that have been identified through projection of introns onto the global alignment of the encoded protein sequences. B) Both species have an intron retention event (IR) but on different positions, which is designated “different position”. C) Both species have an alternative donor event (AD) on the same position (“same type, same position”). D) These events result in the addition of a stretch of amino acids at homologous sites (“homologous modification sites”). E) These events result in similar changes in the protein sequence (”similar modification”).
